# Effect of Perceptual Stress Reduction Control Intervention on the Level of Symptoms in Breast Cancer at Different Time Points

**DOI:** 10.18502/ijph.v49i7.3576

**Published:** 2020-07

**Authors:** Zhongru CAO, Yuting LI, Li WANG, Yanhua LIU, Lei ZHANG, Li MA, Yunfei AN, Yibo WANG, Huiyan LI

**Affiliations:** 1. Department of Medical Oncology, Harbin Medical University Cancer Hospital, Harbin 150040, P.R. China; 2. Ward IV, Department of Breast Surgery, Harbin Medical University Cancer Hospital, Harbin 150040, P.R. China; 3. Nursing Department, Harbin Medical University Cancer Hospital, Harbin 150040, P.R. China

**Keywords:** Bone marrow suppression, Breast cancer, Digestive tract reaction

## Abstract

**Background:**

To investigate the effect of perceptual stress reduction control intervention on the level of symptomatic groups at different time points in breast cancer.

**Methods:**

A total of 124 breast cancer patients were divided into intervention group and control group, 62 cases in each group. Perioperative nursing and chemotherapy nursing were given to the control group, and the intervention group was given the interventional stress reduction control intervention. The level of symptom clusters of different time points were compared between the two groups.

**Results:**

The incidence and severity of myelosuppression in the intervention group were slightly lower than those in the control group. The adverse reactions of bone marrow suppression at T3 were much lower than those in the control group, and the differences were significant (*P*=0.003, *P*=0.043). The control group had higher incidence and more severe symptoms of nausea, vomiting and diarrhea than the intervention group (*P*=0.002, *P*=0.042). The symptoms of breast pain and swelling at T1 in the intervention group were significantly lower than those in the control group (*P*=0.000, *P*=0.000). There was no significant difference in breast symptoms between the two groups at T2 and T3 (*p*>0.05). At the time of T2 and T3 of chemotherapy, the health promotion behavior scores of the intervention group were higher than the control group, and the difference was statistically significant (P_T2_=0.000, P_T3_=0.000).

**Conclusion:**

Perceptual stress reduction control intervention can effectively relieve bone marrow suppression, digestive tract discomfort and breast symptoms, and promote health promotion behavior.

## Introduction

Breast cancer is a malignant tumor of breast epithelial tissue and is one of the common female malignant diseases (1). Surgery and chemotherapy are important methods for the treatment of breast cancer (2). During this treatment, patients may have side effects such as digestive tract reaction, myelosuppression, and cancer fatigue (3).

The theory of perceptual stress reduction control is the result of personal mobilization of self-perception, understanding and behavior to regulate adverse conditions, reduce environmental and other external factors, and obtain hopeful results (4). Both domestic and foreign studies have shown that effective nursing measures can significantly improve the patient’s physical and mental condition, which directly affects the rehabilitation and prognosis of cancer patients (5, 6). Therefore, we explored the effect of conducted perceptual stress reduction control on the symptom group level of breast cancer patients at different time points, in order to improve the physical and mental symptoms during chemotherapy in breast cancer patients and provide a new approach to promote positive healthy behavior.

## Methods

### Research object

A total of 124 patients who underwent breast cancer surgery in Harbin Medical University Cancer Hospital, Harbin, P.R. China from March 2018 to February 2019 were enrolled. Inclusion criteria: patients at 3–4 weeks after surgery; patients who first received chemotherapy (every 3 weeks) after surgery; patients diagnosed as breast cancer by pathological examination; patients with clear consciousness, normal cognitive and sensory function; patients with estimated survival time > 6 months, expected to complete 3 chemotherapy sessions; female patients; patients who were informed and agreed, willing to cooperate.

Exclusion criteria: Patients with inherited diseases such as history of hepatitis B, hyperthyroidism, diabetes, heart disease; patients receiving neoadjuvant chemotherapy before surgery; those with other serious physical diseases and tumors; patients with recurrent breast cancer; patients with history of mental illness or psychological disorder. Overall, 124 patients who met the inclusion criteria were divided into intervention group (2018.9–2019.12) and control group (2018.3–2018.8), 62 cases each. In intervention group, patients have an average age of 43.22±7.08 years, and there were 15 cases with junior high school education and below, 25 cases with high school or secondary school education, 22 cases with junior college education and above. Among them, 37 cases were treated with cyclophosphamide + docetaxel, 11 cases with docetaxel + cyclophosphamide + epirubicin, and 14 cases with epirubicin + cyclophosphamide sequential docetaxel. There were 18 cases in stage I, 34 cases in stage II, and 10 cases in stage III. In control group, patients had an average of 44.62±8.52 years, and there were 14 cases with junior high school education and below, 27 cases with high school or secondary school education, 21 cases with junior college education and above, 37 cases were treated with cyclophosphamide + docetaxel, 10 cases with docetaxel + cyclophosphamide + epirubicin, 15 cases with epirubicin + cyclophosphamide sequential docetaxel. There were 20 cases in stage I, 31 cases instage II, and 11 cases in stage III.

This study was approved by the Ethics Committee of the hospital. The patients were informed and signed the informed consent.

## Procedure

Both groups were given routine perioperative care and chemotherapy care. On this basis, the intervention group was given a perceptual stress reduction control intervention, including.

Feeling stress reduction: 1) dilution of treatment: throughout the perioperative period, chemotherapy process, photos were placed in the patient’s ward wall and bedside; blackboard was hanged in the unit, and the successful cases and the blessings of the patients and relatives were posted on the blackboard; the family members were allowed to place the favorite items or objects without safety risks that the patient was most accustomed to; 2) meditation before going to bed: guiding the patient’s state of mind to enter a state of no thinking before going to bed, guiding the patient to gradually focus on the fine sense of each part of the body, using the adaptive, non-judgmental and non-reactive body awareness of patient to shift stress, anxiety and body pain at the moment. Use “The Tide of the Sea” from the music therapist “Zhengde Music Hypnosis: 365 Nights Deep Sleep” as the background music and guidance, the music was 30 minutes in length, daily before going to bed (21:00–21:30), the steps were: expiration, inspiration-empty, forget, feel and relax.

Cognitive stress reduction: 1) Four-party talks: the primary nurse called the attending doctor, family members to have a conversation with the patient within 2 days of admission, before chemotherapy, before the end of chemotherapy, and the conversation lasted about 30 minutes. The doctors, nurses and family members sat at the patient’s bedside. The conversation was hosted by the responsible nurse. The theme of the three conversations were “controllable factors of breast cancer”. “the key to achieving good chemotherapy results” and “the prognosis of patients with positive mentality and the quality of life”, aiming to explore how to improve patients’ disease cognition, the willingness to accept surgery and chemotherapy, and the benefits of positive attitude of patients towards disease rehabilitation, and to help patients to shift their attention to controllable behavioral responses; doctors were responsible for answering patients and family members’ questions about diseases, treatment methods, feedback on current treatment conditions, and guiding patients to respond to diseases in the most appropriate way. During the conversation, the patient’s emotional state and psychological changes were closely observed. If the patient was found to have low, contradictory emotional performance, transfer or pause the topic, and breathing relaxation and touch relaxation techniques were used to calm the patient’s emotions. 2) Support mutual support counseling: a tutoring was carried out 1 day before discharge, and the psychological counseling volunteers (acquired the national psychologist qualification certificate) conducted group training for the family members, each time 4∼5 family members, the counseling time was about 40 min; the counseling process included four parts: ①establish a supportive relationship: the family members were gathered in the health education classroom, educate nurses, psychological counseling volunteers and family members sat around the round table, each seat had a cup of hot water, a paper towel, paper and pen; ② family members were guided to accept the facts, open discussion on the patient’s feelings of illness and how to get along with the patient after discharge, how to accompany the patient, the family was guided to accept the long-term chemotherapy facts; ③ provide a positive response: the family was guided to list the current things, prioritize and rationalize; help family members to identify patients with negative performance; ④ Rebuild useful ways of thinking, such as positive hospitalize attitudes and regular lifestyles.

Activity stress reduction: 1) the whole-body muscle group elastic exercises: performed once at noon before going to bed every day, following the order of face-neck-shoulders-arms-hands-front chest-back-abdomen-hip-legs-feet, each part was tightened for 10 seconds and then relaxed for 10 seconds. After all parts were completed, repeat from the beginning, 10 minutes a day to achieve the effect of full body relaxation; patient practiced with background music and verbal guidance and followed the sequence and rhythm of the guide; 2) affected limb function exercise: once daily after completed the muscle group elastic exercise, ① The affected side shrugging activity: lying on the bed, the affected side of the forearm was placed on the abdomen, lifting the shoulder as close to the ear as possible for 10 seconds, slowly putting it down, repeating, 10/group, 3 groups/time; ② Affected limb stretching activity: lateral position of the contralateral side was adopted, the upper limb of the affected limb was clamped, the forearm and the upper limb were held at 90 degrees, and the upper body slowly fell backwards. The upper limb and the chest muscle group were stretched to a maximum for 10 seconds and repeated, 10/group, 3 groups/time; ③ Rehabilitation yoga exercise: after the patient had no uncomfortable symptoms, once a day after waking up in the morning, the patient took a cross-legged position, the upper body was straight, and the rehabilitation exercise was: lifted hand pose, extended forward bend, arm lifting pose, sitting eagle pose, embryo in headstand pose, 15∼20 min/time.

### Effect evaluation

Myelosuppression was recorded once every 1 week after the end of the first chemotherapy (T1), the end of the second chemotherapy (T2), and the end of the third chemotherapy (T3). The myelosuppression indexing was performed according to the World Health Organization anti-cancer drug acute and subacute toxicity scores (7): white blood cells, hemoglobin, neutrophils, Platelet, total score: 0 ∼ 16 points. The higher the score, the more severe the inhibition of myelo-suppression.

### Gastrointestinal reaction

The symptoms of nausea, vomiting and diarrhea were recorded at 8:00 pm on the 5th day of T1, T2 and T3, digestive tract response with reference to the 2015 edition of the Ministry of Health “Chinese medicine new drug clinical research guidelines” scoring standard (8): nausea and vomiting, diarrhea, the higher the score, the more severe the bone marrow suppression. Breast symptoms were recorded once at 1 week after T1, T2, and T3, with reference to the scoring criteria for breast cancer breast symptom of the American Chemotherapy Cooperative Organization (9), breast pain, limb swelling, muscle soreness. The 3 symptoms of discomfort were scored using a 4-level scale, “0 points - never, 3 points - always”, score 0 to 9 points, the higher the total score indicated the more severe breast symptoms.

### Health promotion behavior

“Health Promotion Lifestyle Scale” (10) revised by Wenjun Cao was used. This study used this table to evaluate the health promotion behavior of patients during breast cancer treatment. The scale includes 25 items in five dimensions: access information, functional exercise, compliance behavior, emotional adjustment, and health responsibility. Scoring Criteria: using the Likert 5 rating, “0 points - completely unaffected, 4 points - deep affected, the total score was 0–75 points. The higher the score, the more correct the patient’s health promotion behavior. The coefficient of Cronbach’a was 0.74.

### Statistical analysis

The data collected were processed by SPSS20.0 statistical software (Chicago, IL, USA). The quantitative data were expressed as x±s. Analysis of variance of repeated measures was used for comparison between groups. *P*<0.05 for the difference was statistically significant.

## Results

### The comparison of myelosuppressive symptoms at different time points of breast cancer

The severity of myelosuppressive symptoms in the two groups was significantly different between time, group, and time*group interaction (*P=*0.001), the incidence and severity of myelo-suppression in the intervention group was slightly lower than that of the control group at the second chemotherapy. The adverse reactions of bone marrow suppression were much lower than those of the control group at the third chemotherapy, and the differences were significant (*P*=0.003) (Table 1).

**Table 1:** The incidence and severity of myelosuppressive symptoms at different time points during treatment of breast cancer

***Group***	***T1***		***T2***		***T3***		***F_time effect_***	***F_group effect_***	***F_interaction_***
	***Incidence***	***Severity***	***Incidence***	***Severity***	***Incidence***	***Severity***			
intervention	36.81	11.82±1.85	42.76	12.15±2.77	46.57	12.21±2.07	5.334*	7.428*	4.295*
Control	38.95	11.93±1.04	48.78	12.20±2.81	57.25	12.87±2.85			
X2/t	0.296	0.549	0.374	0.813	4.169	2.892			
*P*	0.767	0.658	0.745	0.255	0.003	0.043			

### The comparison of digestive tract reactions at different time points of breast cancer

The severity of digestive tract reaction in the two groups was significant different at time, group, and time*group interaction (*P=*0.001). In the intervention group, gastrointestinal symptoms after the second and third chemotherapy were significantly lighter and the incidence was lower than the control group; after the third chemotherapy (T3), the control group had higher incidence and more severe symptoms of nausea, vomiting and diarrhea than the intervention group, and the differences were statistically significant (*P*=0.002) (Table 2).

**Table 2:** The incidence and severity of digestive tract reactions at different time points during treatment of breast cancer

***Group***	***T1***		***T2***		***T3***		***F_time effect_***	***F_group effect_***	***F_interaction_***
	***Incidence***	***Severity***	***Incidence***	***Severity***	***Incidence***	***Severity***			
Intervention	20.69	4.97±1.02	42.11	2.92±0.80	54.64	1.68±0.74	6.654*	4.892*	4.757*
Control	21.72	4.76±0.73	59.66	4.01±0.86	64.41	3.47±1.02			
X2/t	0.640	0.380	0.374	0.728	4.869	2.992			
*P*	0.487	0.695	0.745	0.367	0.002	0.042			

### The comparison of breast symptoms at different time points of breast cancer

The severity of breast symptoms in the two groups was significantly different between time, group, and time*group interaction (*P=*0.001). The symptoms of breast pain and swelling after the first chemotherapy in the intervention group were significantly lower than those in the control group, and the differences were significant (*P*=0.001). The breast symptoms were alleviated in both groups at the 2^nd^ and 3^rd^ chemotherapy and there was no significant difference between the two groups (Table 3).

**Table 3:** The incidence and severity of breast symptoms at different time points during breast cancer treatment

***Group***	***T1***		***T2***		***T3***		***F_time effect_***	***F_group effect_***	***F_interaction_***
	***Incidence***	***Severity***	***Incidence***	***Severity***	***Incidence***	***Severity***			
Intervention	33.33	1.44±0.53	15.05	1.23±0.50	1.05	1.18±0.37	3.523*	4.419*	4.746*
Control	44.56	3.28±0.78	18.47	1.71±0.71	3.72	1.47±0.70			
X2/t	5.311	5.239	0.838	0.606	0.372	0.417			
*P*	0.001	0.001	0.247	0.473	0.708	0.594			

### Comparison of health promotion behavior scores between the two groups at different time points

The health promotion behavior scores of the two groups were significantly different between time, group, and time*group interaction (*P*=0.001). At the T2 and T3 time points of chemotherapy, the health promotion behavior scores of the intervention group were higher than the control group, and the difference was statistically significant (*P*_T2_=0.000, *P*_T3_=0.000) (Table 4).

**Table 4:** Comparison of health promotion behavior scores between the two groups at different time points

***Group***	***T1***	***T2***	***T3***	***F_time effect_***	***F_group effect_***	***F_interaction_***
Intervention	46.70±8.55	56.95±4.85	62.84±6.22	10.237*	8.640*	12.805*
Control	45.43±7.93	45.16±5.14	56.95±4.85			
*t*	0.485	6.275	11.263			
*P*	0.693	0.000	0.000			

## Discussion

### The effect of perceptual stress reduction control intervention on bone marrow suppression in breast cancer patients during chemotherapy

During postoperative chemotherapy, breast cancer patients will show a variety of symptoms, generally more than two, of which the incidence of bone marrow suppression can be as high as 70% (11). The results in Table 1 showed that the incidence and severity of bone marrow suppression in the control group increased significantly, reaching the highest peak after the third chemotherapy; while in the second and third chemotherapy, the adverse reactions of bone marrow suppression were lower than those in the control group. This result confirms that perceptual stress reduction control intervention can reduce the incidence of myelosuppression during chemotherapy and reduce the severity of myelosuppression. Frederick’s findings found that patients with myelosuppressive symptoms can be improved through activities such as health education, psychological adjustment, and exercise (12).

The theory of perceptual stress reduction control is the result of personal mobilization of self-perception, cognition and behavior to regulate adverse conditions, reduce stress caused by environmental and other external factors, and obtain desired results (13). Based on this, perceptual stress reduction control intervention, as a systematic approach, helps patients adapt to the chemotherapy process, reduce excessive attention to adverse reactions through dilution of treatment, bedtime meditation, especially bedtime meditation effectively reduce physical stress, good sleep can not only enhance the sympathetic nerve excitability to adrenergic receptor-mediated effects in bone marrow cells, but also regulate the migration efficiency of bone marrow stem cells, and stimulate red blood cells, white blood cells proliferation (14,15). The optimistic cognitive basic knowledge makes patients have a sense of control over the disease, and can effectively cope with various problems caused by breast cancer diseases, especially in dealing with adverse reactions of chemotherapy, improve their physiological symptoms to some extent (16,17).

### Effects of perceptual stress reduction control intervention on digestive tract response in patients with breast cancer during chemotherapy

The results in Table 2 show that the incidence and severity of nausea and vomiting and diarrhea in the control group were mainly affected by the number of chemotherapy. The incidence of gastrointestinal symptoms in third chemotherapy was higher and severity was more serious. In the second and third chemotherapy, the symptoms of digestive tract were significantly lighter in the intervention group, indicating that the perceptual stress reduction control intervention in this study can effectively alleviate the degree of adverse reactions in the digestive tract. During the course of chemotherapy, breast cancer releases 5-HT3 to its receptors due to cell death, causing vagal reflexes, leading to significant vomiting reflexes (18.^19^). The literature points out that choosing appropriate nursing methods during chemotherapy and encouraging early activities of patients can promote the recovery of gastrointestinal function (20,21). In this study, the implementation of the four-party talks between doctors and nurses and family members helped patients fully understand the disease, provide patients and their families with a positive response, such as guiding family members to sort out the current needs to do reasonable arrangements for exercise, diet, etc., to promote regular eating, reduce the changes of intestinal microenvironment and enhance the nutrition of patients, thus effectively reducing adverse reactions of digestive tract. It was stated that skeletal muscle-derived IL-6 is one of the potential mechanisms of exercise alleviating chemotherapy-induced digestive tract response, and an individual’s activity program can combat the body damage caused by chemotherapy (22). Therefore, the perceptual stress reduction control intervention guides the whole-body muscle group elastic exercise, the affected limb function exercise, and the rehabilitation yoga exercise to increase the body metabolism, strengthen the body organ function, promote the gastrointestinal motility, and effectively relieve the digestive discomfort.

### Perceptual stress reduction control intervention to reduce breast symptoms in breast cancer patients during chemotherapy

The results of the study showed that after the first intervention, the symptoms of breast pain and swelling in the intervention group were significantly lower than those in the control group. Researchers found that exercise or physical activity not only helps to reduce the symptoms of chemotherapy and digestive tract, but also has a significant effect on breasts and affected limbs of breast cancer patients (23). Based on this point of view, the perceptual stress reduction control intervention exercises the whole-body muscle to the affected limb. It not only relaxes the whole-body muscles, but also reduces the continuous decrease of daily activities after the illness, and the muscle weakness due to the decreased activity endurance. Exercise on the affected side of the limbs was aimed for the affected side of the breast, early use of low-intensity, low-amplitude, small joint movement can relieve joint stiffness, promote blood flow of the affected limb, improve breast swelling without increasing the pain of the limbs (24,25). Yoga exercises can significantly reduce the pain and swelling of breasts in patients with breast cancer (26, 27). Rehabilitation yoga exercise was introduced after the patient had no discomfort, and simple upper limb stretching Yoga exercises were selected.

On one hand, stretching together with soothing music can reduce the pressure on the blood vessels and nerves of the limb brakes, relieve the fatigue state of the muscles (28). On the other hand, pushing and stretching the left and right sides can lengthen and massage the local muscles, helping the body function to recover quickly (29). In addition, the results in Table 3 also showed that there was no significant difference in breast symptoms between the two groups in the second and third chemotherapy groups. The reason may be that breast pain and swelling of the affected limb were caused by surgical trauma, the second and third chemotherapy was far from the operation, thus the pain and swelling decreased significantly.

### Effects of perceptual stress reduction control intervention on health promotion behavior of breast cancer patients during chemotherapy

The results in Table 4 showed that compared with the control group, the health promotion behavior scores of the three chemotherapy groups in the intervention group were higher, indicating that the cognitive stress reduction control intervention can promote the healthy behavior. The perceptual stress reduction control intervention used in this study establishes three stress reduction pathways for support, mutual assistance, knowledge exchange, and activity training among doctors, nurses, families, and patients, and strengthens interaction between the parties so that patients can understand health behavior and disease recovery in depth. The important relationship helps patients to establish a healthy concept, which can effectively control their behavior and form a favorable aspect of disease recovery, so that they can adopt healthier behaviors to promote self-healing (30).

## Conclusion

The perceptual stress reduction control intervention, through feeling stress reduction, cognitive stress reduction and activity stress reduction, can to some extent improve the bone marrow suppression, digestive tract response and breast physiological symptoms during chemotherapy, and help patients to establish a healthy concept and promote self-rehabilitation to the greatest extent.

## Ethical considerations

Ethical issues (Including plagiarism, informed consent, misconduct, data fabrication and/or falsification, double publication and/or submission, redundancy, etc.) have been completely observed by the authors.
